# Relationship Between Inflow Impairment and Skin Oxygen Availability to the Upper Limb During Standardized Arm Abduction in Patients With Suspected Thoracic Outlet Syndrome

**DOI:** 10.3389/fphys.2022.726315

**Published:** 2022-02-11

**Authors:** Jeanne Hersant, Simon Lecoq, Pierre Ramondou, Xavier Papon, Mathieu Feuilloy, Pierre Abraham, Samir Henni

**Affiliations:** ^1^Vascular Medicine, University Hospital, Angers, France; ^2^UMR CNRS 1083 INSERM 6015, LUNAM University, Angers, France; ^3^Service of Thoracic and Vascular Surgery, University Hospital, Angers, France; ^4^School of Electronics (ESEO), Angers, France; ^5^UMR CNRS 6613 LAUM, Le Mans, France; ^6^Sports and Exercise Medicine, University Hospital, Angers, France

**Keywords:** finger pulse plethysmography, transcutaneous oxygen measurement, thoracic oulet syndrome, thoracic outlet compression, ischemia, pathophysiology, arterial inflow, skin blood flow

## Abstract

**Objective:**

Thoracic outlet syndrome (TOS) should be considered of arterial origin only if patients have clinical symptoms that are the result of documented symptomatic ischemia. Simultaneous recording of inflow impairment and forearm ischemia in patients with suspected TOS has never been reported to date. We hypothesized that ischemia would occur in cases of severely impaired inflow, resulting in a non-linear relationship between changes in pulse amplitude (PA) and the estimation of ischemia during provocative attitudinal upper limb positioning.

**Design:**

Prospective single center interventional study.

**Material:**

Fifty-five patients with suspected thoracic outlet syndrome.

**Methods:**

We measured the minimal decrease from rest of transcutaneous oximetry pressure (DROPm) as an estimation of oxygen deficit and arterial pulse photo-plethysmography to measure pulse amplitude changes from rest (PA-change) on both arms during the candlestick phase of a “Ca + Pra” maneuver. “Ca + Pra” is a modified Roos test allowing the estimation of maximal PA-change during the “Pra” phase. We compared the DROPm values between deciles of PA-changes with ANOVA. We then analyzed the relationship between mean PA-change and mean DROPm of each decile with linear and second-degree polynomial (non-linear) models. Results are reported as median [25/75 centiles]. Statistical significance was *p* < 0.05.

**Results:**

DROPm values ranged −11.5 [−22.9/−7.2] and − 12.3 [−23.3/−7.4] mmHg and PA-change ranged 36.4 [4.6/63.8]% and 38.4 [−2.0/62.1]% in the right and left forearms, respectively. The coefficient of determination between median DROPm and median PA-change was *r*^2^ = 0.922 with a second-degree polynomial fitting, but only *r*^2^ = 0.847 with a linear approach.

**Conclusion:**

Oxygen availability was decreased in cases of severe but not moderate attitudinal inflow impairments. Undertaking simultaneous A-PPG and forearm oximetry during the “Ca + Pra” maneuver is an interesting approach for providing objective proof of ischemia in patients with symptoms of TOS suspected of arterial origin.

## Introduction

Although asymptomatic compression of the neuro-vascular bundle during arm movement is found in many apparently healthy subjects, thoracic outlet syndrome (TOS) is considered as a relatively rare disease (Demondion et al., 2006; Burt, 2018; Illig, 2018). According to the standards of the Society for Vascular Surgery, TOS should be considered of arterial origin only if patients have “clinical symptoms due to documented symptomatic ischemia or objective subclavian artery damage caused by compression at the level of the first rib or other related anomalous bone structures” (Illig et al., 2016). Documenting upper limb ischemia is therefore important in confirming the arterial origin of attitudinal symptoms. Indeed, attitudinal compression can result in either incomplete (stenosis) or complete (occlusion) compression of the sub-clavicular artery, and, as a result of these, in different degrees of inflow impairment. To date, the degree of inflow impairment that will likely result in ischemia is unknown, while the presence of ischemia is expected to provide more evidence for the arterial origin of symptoms than the sole presence of inflow impairment.

Digital arterial photo-plethysmography (A-PPG) can be used to estimate inflow changes during attitudinal maneuvers but the changes in pulse amplitude that define inflow impairment remain debated (Gergoudis and Barnes, 1980; Geven et al., 2006; Adam et al., 2018). Transcutaneous oxygen pressure (PtcO2) can estimate oxygen delivery to oxygen consumption mismatch to the forearm (Henni et al., 2019; Abraham et al., 2020) in patients with suspected TOS. We recently showed that the “Ca + Pra” maneuver (a slight modification of the Roos test) allowed quantifiable measurement of the degree of inflow impairment with A-PPG (Hersant et al., 2021), while A-PPG is otherwise a semi-quantitative technique (Moco et al., 2018).

We hypothesized that during the Ca + Pra maneuver, PtcO2 of the forearm would significantly decrease in cases of attitudinal severely impaired arterial inflow, but not where arterial inflow is moderately impaired, as compared to normal inflow. To test this hypothesis, we performed a prospective study in which we recorded inflow changes with A-PPG and forearm ischemia with PtcO2 simultaneously in patients with suspected TOS.

## Materials and Methods

### Experimental Design

All patients that were referred to our laboratory from January 1 2018 to September 30 2021 for the ultrasound investigation of symptoms suggesting the presence of TOS were proposed as participants in the “Systematic Transcutaneous Oximetry Use in Thoracic Outlet Syndrome” (STOUT) study, with the exception of those patients who presented during the successive waves of the COVID-19 epidemic. The STOUT study was designed to allow the simultaneous recording of A-PPG and PtcO2. Recording A-PPG and PtcO2 simultaneously was not possible in the early developments of the tool and only those patients recruited since July 2020 could be analyzed in the present study. After oral and written explanation of the protocol, individual written informed consent was required for inclusion. The protocol was promoted by the university Hospital of Angers and recorded on Clinical trial.gov under reference NCT03355274 before first inclusion. All related procedures were performed in compliance with the principles outlined in the Declaration of Helsinki. As a routine during each visit, we recorded patient demographics and conditions, including age, sex, weight, height, systolic, and diastolic arm pressure on both sides, and ongoing pain-killer treatments. Patients self-completed the “disability of the arm and shoulder” 38-item questionnaire. The score was only calculated if answers to at least 90% of the first 30 questions were available. Ultrasound results were retrieved from patient files and encoded as positive or negative for the presence of an arterial compression during positional maneuvers on each side. Patients refusing the use of their data or unable to understand the information for linguistic or cognitive reasons and patients under 18 years of age were not included in the analysis. The protocol was performed blinded to the results of the investigations performed on the routine visit.

### Provocative Maneuvers

**Figure 1:**
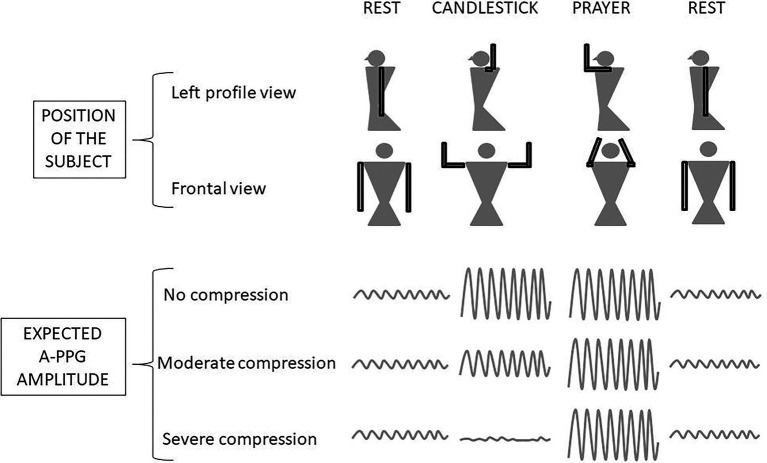
Schematic representation of the Candlestick-Prayer (“Ca + Pra”) procedure and of expected changes in digital arterial photo-plethysmography (A-PPG) pulse amplitude during the different phases. With arm elevation, A-PPG pulse amplitude is expected to increase as a function of hand altitude change relative to heart level. Pulse amplitude is expected to be the same in the candlestick and prayer positions in normal subjects. A decrease of pulse amplitude with arm elevation was considered to be abnormal and the result of a severe arterial attitudinal compression (occlusion or sub-occlusion). Lastly, in cases of partial (mild to moderate) compression, the amplitude observed during the candlestick position remains lower than the one observed in the prayer position.

We previously proposed the candlestick-prayer (“Ca + Pra”) procedure: this is a slightly modified version of the Roos test, during which, after arm elevation, the candlestick/surrender position (“Ca”) is maintained for 30 s (without opening and closing of hands to avoid movement artifacts on A-PPG recordings). After 30 s, a change to the prayer position (“Pra”) with arm elevation with elbows in front of the patient was done and maintained until second 45. At 45 s of the test, upper limb were lowered. Note that, in “Pra” elbow and hands are at the same level relative to heart level as in “Ca” position. The specific interest of “Pra” is to open the costo-clavicular angle and attain arm elevation without vascular compression. Indeed, it has been previously shown that arm elevation results in a physiological increase in pulse amplitude (PA) in normal subjects (Suzuki et al., 1994; Geven et al., 2006; Hickey et al., 2016). Then the “Pra” phase of the maneuver allowed us to confirm that pulse amplitude in the “Ca” was, or was not, already maximal. If not impaired in “Ca,” pulse amplitude would be close to the maximal PA observed during the experiment (generally during the “Pra” phase), while it would be <100% of maximal PA and increase during “Pra” if amplification with arm elevation was moderately impaired during “Ca,” as presented in Figure 1 (Hersant et al., 2021).

### PtcO2 Recordings

**Figure 2:**
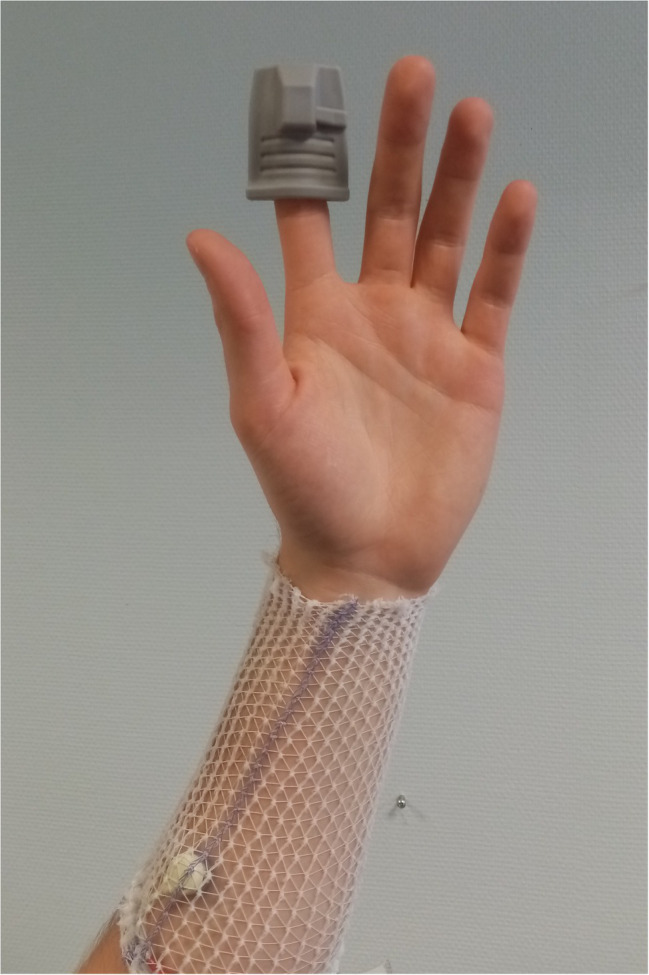
Positions of the photo-plethysmographic probe on the finger and of the transcutaneous oximetry sensor on the forearm of one subject. As shown, a surgifix^®^ net was used to stabilize the sensors and hold the wires in position.

Transcutaneous oxygen pressure (PtcO2) has been used for years at the foot level in patients with suspected critical limb ischemia (Constans et al., 2019) or during exercise to provide quantitative results of regional ischemia (Abraham et al., 2018). We recently showed that during attitudinal maneuvers in TOS, the impaired perfusion to the upper limb resulting in forearm ischemia can be estimated bilaterally and simultaneously with PtcO2 (Henni et al., 2019; Abraham et al., 2020). PtcO2 recordings were performed with TCM400 (Radiometer, DK) with three E5250 probes. Double calibration to air was performed before each recording session. Once the system was calibrated, probes were positioned on the dorsal aspect of each distal third of the forearm (Figure 2), with the patient standing still. The third probe was used as a reference on the neck at the level of first thoracic vertebrae. After probe positioning a period of 15–20 min was observed in which the skin was heated to 44°C and stable calibration values were attained. All tests started by a minimum of 30 s of recording at rest in the standing position. The recording of PtcO2 absolute values was undertaken with a PtcO2 software that automatically calculates the decrease from rest of oxygen pressure (DROP) values. DROP corresponds to the subtraction of the chest-PtcO2 changes from rest to the upper limb-PtcO2 changes from rest. The resulting DROP is expressed in millimeters of mercury (mmHg) and allows us to get rid of the error due to the unpredictable transcutaneous gradient (Abraham et al., 2003; Henni et al., 2018). DROP is reliable for intra-test and test–retest recordings (Henni et al., 2018). For the analysis, the minimal forearm DROP value (DROPm) during or following the “Ca + Pra” maneuver was recorded. Note that due to the change in probe altitude relative to heart level a physiological decrease of 5–10 mmHg of DROP value was expected in the absence of arterial inflow impairment (Eickhoff and Jacobsen, 1982).

### Photo-Plethysmography Recordings

Arterial pulse photo-plethysmography (A-PPG) allows bilateral automatic recordable measurements (Sallstrom and Thulesius, 1982; Colon and Westdorp, 1988; Lai et al., 1995; Geven et al., 2006; Chen et al., 2014). We recorded arterial pulse photo-plethysmography (A-PPG) at the second finger of both hands using adult finger soft-tip SpO2 sensors (Sino-K, CN) with a home-made recording program that allows A-PPG recording on a 50 Hz basis (Figure 2). The recording was started at least 10 s before the start of the provocative maneuver and stopped at least 1 min after the end of the provocative maneuver. Each recording allows the detection of the A-PPG signal during each cardiac cycle. Arterial pulse amplitude (PA) was determined as the distance between the maximal and minimal A-PPG value over 1.5 s. Moving averaging was done on 10 points. A transient artefact peak was systematically observed at upper limb elevation and lowering. Then, PA at rest, during “Ca,” and “Pra” phases were calculated between 10 and 5 s before, between 5 and 20 s after the start of the “Ca” maneuver, and between 35 and 40 s (5 s after the start of the “Pra” maneuver) to avoid the movement artefact influence over recorded results. Since arm elevation results in a physiological increase in PA in normal subjects (Suzuki et al., 1994; Geven et al., 2006; Hickey et al., 2016), we normalized PA as a percentage of the highest PA observed during “Pra” or “Ca.” For the comparison with DROPm results, PA changes (PA-change) were expressed as the difference between PA during “Ca” and PA observed at rest.

### Statistical Analysis

Kolmogorov–Smirnov tests were used to test the distribution of variables and results are presented as mean +/− standard deviation (SD) for parametric continuous variables or median [25th/75th centiles] for non-parametric continuous variables. For the simultaneous A-PPG and PtcO2 recording, we aimed to compare the DROPm results as a function of PA-changes. Thereby we calculated the median DROPm value for each decile of PA-change. Then, we analyzed the relationship between median values of PA-change and median DROPm within each decile with linear and second-degree polynomial (non-linear) models. ANOVA with two-sided Dunnett post-test was used to compare DROPm values in each centile of PA-Change to the DROPm value observed in the group with the highest PA-change results. All statistical analyses were performed using SPSS (IBM SPSS statistics V15.0, Chicago, IL, United States). For all tests, a two-tailed *p* < 0.05 was considered to be statistically significant.

## Results

We studied 110 arms of 55 different patients (22 males and 33 females). Patients were 25.9 ± 13.3 years old, with weight 73.8 ± 13.5 kg, and height 170 ± 9 cm. Systolic and diastolic pressures were 129 ± 14 and 79 ± 9 mmHg on the right side and 128 ± 17 and 81 ± 13 mmHg on the left side with no patient showing a difference in arm pressure in excess of 20 mmHg. All but five of the patients were right-handed. The DASH score of the subjects was 35 ± 24% with nine missing scores. Twenty-four were off work, 17 of whom were not working because of their upper limb pain. Twenty-four patients had right unilateral, 23 left unilateral, six bilateral pain or discomfort, and two reported no pain. Twenty-five took pain killers on a regular basis because of pain or discomfort. Positional tests during their medical routine visit reproduced usual symptoms in all but 14 of the patients. Thirty-seven patients had a positive ultrasound result on one or both sides.

**Figure 3:**
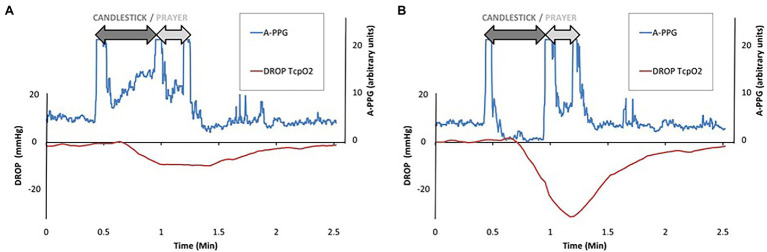
Recordings of a patient with unilateral left symptoms. Note that raw values are for pulse amplitude of photo-plethysmography (A-PPG) and are expressed in arbitrary units. As shown, pulse amplitude increased on the right arm (left panel “**A**”) while it decreased on the left arm (right panel “**B**”) during the candlestick position. As a consequence, the DROP value was lower on the left than on the right side. Note that, due to the relatively slow half-time response for PtcO2, the minimal DROP value (−29 mmHg) is observed while the pulse amplitude is already restored on the left side.

A typical example of simultaneous A-PPG and PtcO2 recording in a patient with unilateral symptoms is presented in Figure 3. Note that the minimal value is reached while pulsatility is already restored due to the relatively slow response of PtcO2.

**Figure 4:**
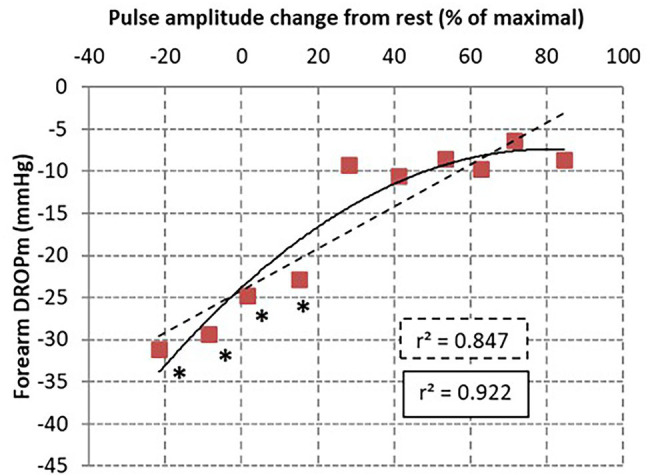
Scatterplot of DROPm results from transcutaneous oxygen pressure recordings for each decile of pulse amplitude (PA) changes in the 110 arms tested. The relationship was tested with a linear (dotted line) and second degree polynomial models. As shown, the highest coefficient of determinations was found for the non-linear model. * is *p* < 0.05 from the highest centile.

DROPm values ranged −11.5 [−22.9/−7.2] and − 12.3 [−23.3/−7.4] mmHg on the right and left forearms, respectively. Resting PA was 25.9 ± 13.3% on the right side and 29.2 ± 16.3% on the left side. PA-change ranged 36.4 [4.6/63.8]% and 38.4 [−2.0/62.1]% in the right and left forearms. When splitting the PA-changed in deciles (11 observation per decile), the relationship between median DROPm values and median PA-changes was *r*^2^ = 0.922 with a second-degree polynomial model but only *r*^2^ = 0.0.847 with a linear approach, as presented in Figure 4. As shown, the decrease in DROPm was approximately 10 mmHg when median change in PA exceeded 20% (which means that it increased on the average by 50% of resting values). This 10 mmHg decrease was the one expected from altitudinal change of the probe relative to heart level. As shown in Figure 4, ANOVA confirmed that a difference from the highest centile of PA-change reached significance only for the four lowest centiles.

## Discussion

For years, a number of authors have argued that neurogenic TOS comprises over 90% of all TOS cases, while arterial TOS is rare (Sanders et al., 2007; Illig et al., 2016). Obviously, there is an overlap between these two forms and many patients with TOS have intricate signs of arterial and neurogenic compression (Likes et al., 2014; Illig et al., 2016; Ferrante and Ferrante, 2017), underlining the interest of improving arterial investigations in patients with suspected TOS (Molina and D’Cunha, 2008; Likes et al., 2014).

Ultrasound allows recordings of arterial inflow and remains relatively simple (Sobey et al., 1993; Nord et al., 2008). Nevertheless, it is a manual technique, it can only measure one side at a time, and it does not provide evidence of ischemia itself. Arterial pulse amplitude (PA) estimation by finger A-PPG allows bilateral recordings and is an attractive tool in the context of arterial inflow impairment that allows objective and simultaneous recording of inflow throughout the provocative test (Colon and Westdorp, 1988; Geven et al., 2006). Raising the arm above heart level changes transmural pressure and increases the systolic amplitude of the finger pulse amplitude in normal subjects (Suzuki et al., 1994; Jagomagi et al., 2005; Hickey et al., 2016). Because photo-plethysmography is a semi-quantitative technique which, to date, lacks an objective proof of the expected normal increase at the individual level, various definitions of inflow impairment during A-PPG are used (Gergoudis and Barnes, 1980; Geven et al., 2006; Adam et al., 2018).

We previously proposed the “Ca + Pra” maneuver as a way of normalizing PA changes on A-PPG during abduction tests and provide quantitative results from this otherwise semi-quantitative technique. Since the normal response of A-PPG is an increase in amplitude with arm elevation, it is likely that any PA decrease from rest is an abnormal value unmasking a severe compression as proposed by Adam (Adam et al., 2018). Nevertheless, even an increase in PA can indicate the presence of arterial inflow impairment. Indeed, if the PA observed during the “Ca” is lower than the maximal PA (generally observed during the “Pra” phase), it means the PA amplification was blunted during abduction and external rotation (as a result of mild to moderate arterial positional compression).

It is of interest to note that classical methods used to estimate inflow impairment (ultrasound, angiography, or arterial photo-plethysmography) provide no evidence of the presence of ischemia. Indeed, sufficient perfusion can persist despite arterial positional non-occlusive compression, resulting in no significant oxygen consumption/delivery mismatch. There are many potential advantages of using PtcO2 in TOS. It allows direct monitoring of oxygen availability as the determinant of pain and of the decline in force during ischemia (Hogan et al., 1999). The specific interest in the present study was the ability to objectively monitor both arms throughout the period of, and in the recovery period from, the provocative tests. Further, associating A-PPG and PtcO2 provided unique original results in the present study. As for any arterial lesions, a mild to moderate stenosis is expected to have no or little consequences on oxygen delivery.

As for any kind of arterial lesion that remains hemodynamically non-significant if not severe enough, we did not expect the relationship between DROPm and changes in PA to be linear. As shown in Figure 4, DROPm decreased with the decreased A-PPG amplitude but rather followed a non-linear relationship. This could be an explanation for the false negative results observed in our previous studies when comparing PtcO2 to arteriography (Abraham et al., 2020). On the other hand, the “normal” increase in PA-change (PA close to the maximal recorded PA) or “impaired” increase in PA-change (PA on the “Ca” lower than maximal PA that is assumed to reflect the presence of an arterial compression) resulted in only a limited decrease in DROPm value, as previously discussed. This moderate DROPm decrease is probably the result of local change in hydrostatic pressure (Dooley et al., 1997), while the altitude of the limb probe is changed as compared to the chest reference electrode used for the calculation of the DROPm. Previous results reported a PtcO2 decreased with foot elevation at an average rate of 1.2% per millimeter Hg change in mean arterial blood pressure (Eickhoff and Jacobsen, 1982). Similarly, extrapolation of the results of Blake et al. suggested a 1.5–2 mmHg decrease in PtcO2 for each 10 cm limb elevation above resting level in normal subjects (Blake et al., 2018). Then, the average 10 mmHg decrease in DROPm that we found in patients with PA close to 100% was expected as a result of hydrostatic pressure changes only.

Finally, from a clinical point of view, it is unlikely that an arterial compression that induces no ischemia could be the cause of upper limb symptoms of arterial origin. We believe that it is important because, PtcO2 estimates ischemia and provides unique evidence for a causal relationship between the arterial compression and the symptoms. In the absence of ischemia, the presence of arterial compression is an indirect indication that a neural compression is likely present and possibly responsible for the symptoms. From a technical point of view, the simultaneous analysis of both arms is intriguing, as it would allow for direct, real-time comparison of TOS between arms, removing potential time-dependent sources of error. Such time-dependent sources of error are probably the cause of the large number of inconsistencies that were found between PtcO2 and PPG results, and the results of ultrasound imaging.

There are limitations to the present work. Firstly, we did not measure the presence or absence of venous compression in our patients. This is an issue because it was shown that PtcO2 measurements significantly decrease during 10 min of venous congestion induced by tourniquet venous occlusion (Geis et al., 2008). We cannot therefore exclude the possibility that some of the low DROPm values observed in the absence of impaired A-PPG amplitude resulted from underlying venous outflow impairment.

Secondly, we do not report, and do not compare our results to, ultrasound imaging or angiography in our patients. Radiological imaging is lacking in most of our patients. All patients had ultrasound, but as previously discussed, measurements were not taken simultaneously, with ultrasound (sometimes) performed in the recumbent position while PtcO2 and A-PPG recordings were taken in the standing position. Patient position significantly interferes with the ability to unmask arterial compression during attitudinal tests (Cornelis et al., 2008), and difference in position is likely one of the reasons for the wide discrepancies that we previously observed when comparing the results of ultrasounds, arteriography and PtcO2 (Abraham et al., 2020).

Thirdly, correlation with clinical symptoms was not the primary aim of the present study, but it warrants future investigation. In the light of the relationship between A-PPG and PtcO2, it is possible that positive imaging or clinical investigations (auscultation, pulse palpation, etc.) do not necessarily result in ischemia. This could provide an explanation for the apparent high rate of “false positive” results for these investigations observed in healthy subjects (Plewa and Delinger, 1998; Nord et al., 2008).

Fourthly, the “Ca + Pra” procedure is limited to 30 s of abduction. It could be considered an issue and suggested as a cause of negative results, with the idea that longer period of moderate inflow impairment would have resulted in ischemia. On the contrary, we believe that it is of interest because the duration of abduction is comparable in all our subjects while abductions of different durations would not allow subject-to-subject comparisons. One important point here is to keep in mind that although PtcO2 responds relatively slowly (the 90% response to any abrupt change is approximately 20–30 s), the duration of the recording period is long enough to have DROPm reach a minimum and return to baseline value before the end of the recording.

Last, the number of patients that we studied remains small and investigations in a larger group are required to improve the generalizability of our results and/or allow sub-group specific analyses (such as by gender or age).

## Conclusion and Perspectives

To the best of our knowledge, this is the first report of simultaneous recordings of PtcO2 and A-PPG, revealing a non-linear relationship between inflow changes and oxygen availability in patients with suspected TOS. This result underlines the fact that various degrees of positional arterial compressions may occur, and that defining whether an arterial compression occurs during positional tests should probably not result in a binary “Yes/No” response. Last, a physiological decrease in DROPm (ranging 5–15 mmHg) with limb elevation is to be accounted for in the interpretation of PtcO2 results.

From a physiological point of view, the “Ca + Pra” maneuver appears to us an interesting approach to facilitate the interpretation of A-PPG results in TOS. Using this test, a measurable ischemia (estimated through PtcO2 decrease) is observed only in cases of decreased pulse amplitude compared to rest (assumed to result from arterial occlusion or sub-occlusion) during the attitudinal maneuver. Although the present results observed with A-PPG should be confirmed with simultaneous PtcO2 and ultrasound recordings, we advocate the position that inflow impairment observed during arm abduction is not necessarily sufficient to induce forearm ischemia and should not be used alone to argue for an arterial classification of TOS.

From a clinical point of view, the simultaneous use of A-PPG and PtcO2 during a “Ca + Pra” maneuver might be critical interest to prove not only the presence of a compression (arterial impaired inflow) but also the presence of ischemia, as underlined by recommendations. We believe that the recorded evidence of both inflow impairment and ischemia could be essential in cases of conflict between the patients and their surgeons, or to explain why subjects might become asymptomatic after kinesiology and reeducation despite persistent compression at imaging.

## Data Availability Statement

The raw data supporting the conclusions of this article will be made available by the authors, without undue reservation.

## Ethics Statement

The studies involving human participants were reviewed and approved by CPP-Ile de France VII. The patients/participants provided their written informed consent to participate in this study.

## Author Contributions

JH, PR, PA, and SH: design and conception. MF: technical support. PA and SH: administrative process and funding. JH, PR, SL, XP, MF, PA, and SH: data acquisition and final approval. JH, PR, MF, PA, and SH: data analysis. PA: drafting manuscript. JH, PR, SL, XP, MF, and SH: critical comment to the draft. JH, PR, PA, and SH: statistical analysis. All authors contributed to the article and approved the submitted version.

## Funding

The University Hospital of Angers and Angers Technopole partly funded the project.

## Conflict of Interest

PA is the beneficiary of support from the Radiometer®, Perimed®, and Medicap® companies. None of these companies interfered with the present project.

The remaining authors declare that the research was conducted in the absence of any commercial or financial relationships that could be construed as a potential conflict of interest.

## Publisher’s Note

All claims expressed in this article are solely those of the authors and do not necessarily represent those of their affiliated organizations, or those of the publisher, the editors and the reviewers. Any product that may be evaluated in this article, or claim that may be made by its manufacturer, is not guaranteed or endorsed by the publisher.
